# Role of family in supporting children with mental disorders in Qatar

**DOI:** 10.1016/j.heliyon.2023.e18914

**Published:** 2023-08-09

**Authors:** Abdulnaser A. Fakhrou, Taha R. Adawi, Sara A. Ghareeb, Atef M. Elsherbiny, Maryam M. AlFalasi

**Affiliations:** Qatar University, College of Basic Education, Qatar

**Keywords:** Mental disorders, Children, Family

## Abstract

**Background:**

Mental disorders can significantly impact children's lives and affect their emotional, cognitive, and behavioral development. Family support and care is critical to the well-being of children, particularly children with mental disorders. However, given the “gap” between research and practice“,” there have been very few studies in the Arab region that focus on the role of the family in supporting children with mental disorders. The study also examines how families cope with caring for a person with mental disorders and what behaviors may influence the patient's distress. In addition, the study will examine the importance of family rehabilitation and integration of people with mental disorders into society.

**Methods:**

The study adopts the descriptive-analytical method and uses a questionnaire to gather data from the participants. The 350-parents sample (with 113 boys, 237 girls) was selected from the Shafallah Center for Integrating People with Disability. Morgan's law is used to determine the sample size.

**Results:**

The results show that there are statistically significant differences in the role of family members in supporting people with mental disorders due to two variables: Gender and Work. Age has no statistically significant effect on the role of family members in supporting people with mental disorders.

**Conclusion:**

This study is the first study conducted to investigate the role of family in supporting children with mental disorders in the Gulf Cooperation Council (GCC) in general and Qatar in particular. The results show that families should cope with the needs of a person with a mental disorder. Family rehabilitation is important in the care of people with mental disorders. There are certain behaviors of family members that can increase or decrease stress for the person. The results suggest that the family plays an essential role in supporting and promoting the lives of people with mental disorders and recommending effective ways to cope with them.

## 1 Introduction

Mental disorders can significantly impact the lives of children, affecting their emotional, cognitive, and behavioral development. The support and care of families are crucial for children's well-being, especially those with mental disorders. According to a report by the World Health Organization (WHO), “families are essential partners in mental health care [1] ". Family members play a vital role in providing emotional support, helping children access appropriate treatment, and advocating for their needs within the healthcare system. In this paper, we will review the current literature on the role of families in supporting children with mental disorders and the strategies they can use to enhance their children's well-being.

The World Health Organization states that approximately 450 million people have been diagnosed with mental disorders [1]. The prevalence of mental disorders has increased dramatically; approximately 1 in 6 people worldwide suffer from mental disorders. Mental disorders occur in both men and women, with anxiety, depression, bipolar disorder, and eating disorders being more common in women than in men [2,3]. The previous statistical article by Ritchie and Roser [4] shows that 792 million people were diagnosed with mental disorders in 2018. Mental disorders affect not only the patient but also the family members.

Family support has been recognized as an essential factor in the mental health care of children with mental disorders. Current research examines the role of families in supporting children with mental disorders and the strategies they can use to improve their children's well-being.

Families also play a critical role in helping children access appropriate treatment for their mental disorders. Family members can advocate for their children's needs in the health care system, help them adhere to treatment plans, and monitor their progress [5]. In addition, family involvement in the treatment process has been shown to improve treatment outcomes for children with mental disorders [6]. For example, family-based interventions such as family therapy have been shown to reduce symptoms of mental disorders in children and improve family functioning [7,8]. In addition to providing emotional support and helping children access appropriate treatment, families can use various strategies to enhance their children's well-being. One such strategy is education. Family members can learn about their children's mental disorders, the treatments available, and the resources available to them. This can help them better understand their children's needs and how to best support them [9].

Another strategy is to strengthen children's resilience. Family members can help children develop coping skills, such as problem solving, emotion regulation, and social skills, which can help them better manage their mental disorders and the challenges they face [10]. In addition, family members can help their children identify their strengths and interests, which can help them develop a sense of identity and purpose [11].

A study conducted by Bener et al. [12] examined the prevalence and burden of common mental disorders in the Qatari population. The study shows that the prevalence of mental disorders is higher in women than in men. The results of the study indicate that there is an urgent need for further research to promote the community of people with mental disorders and improve their lives.

Another study conducted by Ghuloum and Bener [13] attempted to determine the prevalence of the most common mental disorders among the Qatari population attending a health facility. Personal interviews and a questionnaire were used. The results of the study show that the prevalence of mental disorders is 36.6%, with depression being the most common mental disorder at 13.5%, followed by anxiety. Qatari women are at higher risk for mental disorders than men, and a quarter of the Qatari population who visited a health facility suffered from at least one disorder. The findings of Eaton et al. [14] show that self-stigma is prevalent among parents of children with mental disorders, which is a social burden that leads to a decreased sense of caring.

In summary, families play an important role in supporting children with mental disorders. Through emotional support, advocacy, and various strategies, families can increase their children's well-being and improve their mental health. It is important that health care providers recognize and value the role of families in caring for children with mental disorders and provide them with the resources and support they need to effectively fulfill their role. The current research aims to answer the following questions:The study aims to answer the following questions:

1) What is the role of the family in supporting people with mental disorders?
2) Which needs of a person with a mental disorder does the family need to cope with?
3) What is the importance of family preparedness in caring for a patient with a mental disorder?
4) What are the behaviors of family members that may increase or decrease stress for the patient?
5) Are there statistically significant differences in the role of family members involved in supporting people with mental disorders due to the three variables: age, gender, and work?

Although there are many studies focusing on individuals with mental disorders, there is a lack of research on the role of the family in supporting patients with mental disorders. This study aims to fill the gap in this area, taking into account the specific context of Arab culture, where mental disorders are stigmatized.

## 2 Literature review

### 2.1 The importance of understanding the mental disorder and persons with mental disorder behavior

The first step for a family to support their patient is to learn about their patient's case. Without knowing the nature of the disease, it will not be easy to support him in an effective way. For example, without education, it is difficult for the family to understand or appreciate the severe symptoms of mental disorders, such as the troubling thoughts associated with schizophrenia or the suicidal tendencies associated with depression. The family should understand that any bizarre behaviors of the person with mental disorders are considered manifestations of the disorder and not intentional actions [15,16]. To avoid frustrations and tensions, it is urgent to educate the families of people with mental disorders. Educating families and involving them in the treatment process reduces the symptoms of the disorder, the number of hospital days, and the number of relapses.

The family is considered a partner in mental disorder recovery because they are responsible for the diagnosis of the person with a mental disorder, as the person with mental disorders spends a lot of time with their family. She can infer unusual behaviors of the patient. In addition, it can promote the person's daily living skills and self-efficacy. The family is responsible for preparing and adapting the home to help the patient improve his skills and abilities [17].

One of the most important roles of families is to provide emotional support to their children. Emotional support from family members has been associated with better mental health outcomes in children with mental disorders [18,19]. Family members can provide children with a safe and supportive environment in which to express their feelings, which may help reduce symptoms of anxiety and depression [20]. In addition, family members can reassure and encourage their children, which may increase their self-confidence and self-esteem [21].

### 2.2 The behaviors of the families may increase or decrease stress for people with mental disorders

People with mental disorders need the compassion, love and encouragement of their families; they need to feel that they are not alone. Feeling loved, respected, and supported does wonders for helping them find healing. Piat et al. [22] claim that people with mental disorders also need affection, a sense of belonging, and a feeling of being surrounded by love. They also need to feel how proud their family members are of their progress.

Social interaction plays a central role in the recovery process. Young and Ensing [23] point out that contact with family members, friends, and the community is a clear indicator that the person with mental disorders has recovered and is functioning as usual. In addition, family can accelerate a person's recovery by instilling hope and readiness in the person with a mental disorder [24].

On the other hand, there are some behaviors that can exacerbate the symptoms of a mental disorder. Families who express criticism and hostility toward patients are likely to increase relapse. In addition, psychological or physical abuse can negatively affect the recovery of a person with mental disorders [25].

Family members usually care for the person with mental disorders, although they are frustrated because of the lack of progress. They do not realize that certain behaviors are part of the disorder and that there is no urgent treatment for a mental disorder. They find it painful to live with people who have been diagnosed with a mental disorder [26].

Critical remarks also have a negative effect on the patient, such as saying that the patient is lazy, because the tone of voice may refer to criticism. In addition, excessive caring is a central cause that can affect the patient, such as treating the person with mental disorders like a child and doing the person's affairs in his or her place [27].

### 2.3 Including people with mental disorders in society

The inclusion of people with mental disorders in society requires the constant cooperation of family members with therapists and teachers in programs offered by the institutions that provide care and integration of people with mental disorders in society. In addition, parents should involve the person with a mental disorder in self-care as much as possible and encourage them to participate in society and engage in their local community as much as possible [28].

While social inclusion is considered a human right for all people, the integration of people with mental disorders is essential for patient recovery. Hall et al. [29], in their study entitled “Social inclusion and exclusion of people with mental illness in Timor-Leste: a qualitative investigation with multiple stakeholders,” examined the social experiences of people with mental disorders and their families. A sample of eighty-five participants was selected to be interviewed. The purpose of the study is to examine the social inclusion of people with mental disorders and their families in Timor-Leste. The results of the study show that people with mental disorders are stigmatized because they are considered dangerous, stupid, and unpredictable. They are also exposed to bullying, sexual abuse, and physical violence. In addition, they face various barriers to social services, such as employment, education, land ownership, marriage, and denial of the right to vote.

Bessa and Waidman [30] conducted a study aimed at investigating the needs of family caregivers caring for a person with mental disorders. The results of the study showed that family members caring for a person with a mental disorder need financial support, adjustment after the diagnosis of pathology, hospitalization, family comfort, and involvement in social services. The family needs to adjust its life routine to cope with its new activities in caring for the patient. It also needs better guidance to deal with the problematic behaviors of a person with mental disorders. This support and guidance reduces the burden on the family.

Peer-to-peer training is essential for helping families cope with mental disorders. Respect, responsibility, support, and empathetic understanding of others’ situations are essential principles of peer-to-peer support [[31], [32], [33]] Peer support trainings are based on the qualifications of people who have experienced and successfully managed mental disorders. They can offer support, hope, and encouragement to other families as they have faced the same circumstances. It helps reduce stress and worry and reinforces self-determination and knowledge about mental disorders [34].

In addition, parent training is more effective in helping parents with possible interventions and tools to increase patient competence. A wide range of training and interventions have been developed for parents of children with special needs, including “P Triple Stones Stepping”. It is part of Australia's Triple P, Positive Parenting Program, which aims to train parents on how to improve their patients' social behavior. It is a program that successfully prepares parents of people with mental disorders to participate in parenting and guiding others. This leads to a reduction in parents' stress levels and an improvement in their ability to adapt. It tends to reduce or eliminate acute behavioral, emotional, or developmental problems of people with mental disorders (Kleefman et al., 2014). [35].

### 2.4 The impact of mental disorder on the family

The needs of the family often go unrecognized. Family members are under constant stress and feel that they themselves need emotional support and perhaps psychological care and treatment. Their loved one's illness can lead to discrimination, permanent financial burden, and unemployment due to caregiving responsibilities.

Living with and dealing with people with mental disorders is ultimately challenging. Families struggle for years to find information and support. Numerous studies indicate that parents of a child with a mental disorder often exhibit high levels of tension and stress when it comes to meeting their child's needs [[36], [37], [38], [39], [40]]. They also feel helpless because of certain government policies and fear for their children's uncertain future. Harnois et al. [41] point out that family members of people with mental disorders face challenges in the workplace, such as lower productivity, higher error rate, lower decision-making ability, burnout, tension and conflict with other workers, and loss of motivation. Therefore, the needs of parents and ways to support them should be considered. The role of the family is considered the cornerstone of support, treatment, and recovery for individuals diagnosed with mental disorders. Families are considered the primary caregivers tasked with promoting patients' mental health and well-being [42,43]. The family represents holistic support for its members; it encompasses all aspects of the patient's life and assumes responsibility for day-to-day care. The family oversees and monitors all behavioral and developmental changes that occur in the person with mental disorders. They can also assist professionals with important information about the strengths and weaknesses of the person with mental disorders [17]. Recent studies in Qatar emphasize the central role of families in supporting children and improving quality of life, especially for people with disabilities [44,45].

Family members pay a high price for caring for a person with mental disorders; they face financial problems, stigma, coping problems, and psychological morbidity. Caring for a person with mental disorders is also a burden. According to Hoenig and Hamilton [46], the burden of caregiving has two different aspects: objective burden and subjective burden. The objective burden represents the measurable consequences that affect family members; these include an economic burden, interruptions, loss of leisure time, and rigorous mobilization. Subjective burden, on the other hand, is the impact of the mental disorder on the psychological well-being of family members, including internal emotions and feelings such as depression, lostness, sadness, guilt, and shame. Mental disorders have a serious impact on families and change the way they live their lives. They lead to tension, stress, troubling feelings, and uncertainty.

Family members experience constant anxiety, confusion, stress, shame, self-blame, guilt, fear, and sadness. These feelings vary over time depending on how far the disorder has progressed and how prepared family members are for it. Over time, family members experience changes and cope [23].

### 2.5 Mental disorder as a stigma

Family members of a person with a mental disorder view the disorder as a stigma; they view the existence of a person with a mental disorder as a punishment from God. Feelings of shame, contagion, and guilt are common among family members. These feelings can lead to isolation and avoidance of contact with friends and neighbors. Blame is usually associated with poor parenting skills and adverse experiences. In addition, feelings of guilt decrease self-esteem and self-confidence. Stigma can prevent people from accessing health and education services. It also affects the chances of marriage for a person with mental disorders [47,48]. Stigma and discrimination are considered intrinsic barriers to the inclusion of people with mental disorders in society. Consequently, their voices go unheard.

## 3 The present study

Despite the importance of family support for children with mental disorders, there is limited research on the specific roles and strategies families can use to effectively support their children's mental health needs. To date, there are few studies that directly address mental disorders in children in the Arab world and in the Gulf Cooperation Council (GCC) in particular [49]. Such empirical work is essential to examine and understand the context of mental disorders. This study aims to fill this gap by examining the role of families in supporting children with mental disorders.

## 4 Methods

### 4.1 Participants and procedures

The first author contacted the director of the Shafallah Center for the Integration of Persons with Disabilities and learned that the purpose of the study was to survey parents of children with mental disorders about their perceptions of mental health needs and practices. A total of 750 parents were contacted by phone or email to confirm their participation. Morgan's law is used to determine the sample size. A total of 350 Qatari mothers and fathers (M = 37.6 years, SD = 6.5 years) of children with mental disorders (113 boys, 237 girls; M = 6.3, SD = 4.9) volunteered to participate in the survey and signed an informed consent form. A response rate of at least 40% has been documented for accurate, reliable data (Kramer et al., 2009) [50]. Children were diagnosed with the following: 98 with anxiety disorders, 107 with depression, 88 with attention-deficit/hyperactivity disorder (ADHD), and 57 with conduct disorder. The children were diagnosed at Hamad Medical Corporation (HMC), the main public health care provider in Qatar, using the Diagnostic and Statistical Manual of Mental Disorders (DSM-5) criteria [51]. Ethical approval was obtained from Qatar University's IRB committee (QU-IRB 1529-EA/21), which required a consent approval from all participants.

The software IBM SPSS v26 and MPLUS v7 were used for data analysis. Frequencies and percentages, means and standard deviations, person correlation and Cronbach's alpha were used. Equation of the range as the following: ((1: 1.79) strongly disagree, (1.8 : 2.59) disagree, (2.6 : 3.39) neutral, (3.4 : 4.19) agree, (4.2 : 5) strongly agree).

### 4.2 Characteristics of the study sample

The frequencies and percentages of the sample are calculated according to the variables gender, age, and occupation.

Table (1) shows the distribution of the sample members by gender as the following:

• (58%) of the study sample are females and (42%) of the sample study are males.
• (37.1%) of the study sample their ages between 30 years and 45 years, (30.9%) of the study sample their ages less than 30 years, and (32%) of the study sample their ages more than 45 years.
• (58.9%) of the study sample are employed and (41.1%) of the study sample are unemployed.

**Table (1):** Distribution of sample members by gender.

Variable	Category	Frequency	Percent
Gender	Male	147	42%
	Female	203	58%
Age	Less than 30 years	108	30.9%
	Between 30 years and 45 years	130	37.1%
	More than 45 years	112	32%
Occupation	Employed	206	58.9%
	Unemployed	144	41.1%

### 4.3 Study instrument

#### 4.3.1 Instrument construction

After reviewing previous studies on the subject of the investigation, a questionnaire was developed that contained two main parts: The first part consists of demographic data about the research sample, and the second part consists of 4 main dimensions that serve the purpose of the study:

**The first dimension:** the role of the family in supporting people with mental disorders consisted of (7 items).

**The second dimension:** the family must cope with the needs of a person with a mental disorder consists of (7 items).

**The third dimension:** the importance of family rehabilitation in caring for a patient with a mental disorder consists of (6 items).

**The fourth dimension:** the behaviors of family members that may increase or decrease stress for the patient consist of (5 items).

This questionnaire uses Likert scale ranging from 1 (strongly disagree) to 5 (strongly agree). Concurrent validity was supported.

#### 4.3.2 Face validity

The preliminary version of this tool was circulated to an advisory committee for feedback. A four-member committee was asked to evaluate the overall format, domains, and items of the questionnaire. The committee was comprised of a professor and associate professor of mental health counseling, an investigator specialized in children mental health disorders and a psychiatrist. Iterative feedback was used to revise the questionnaire and then submitted to an expert with PhD degree in Shafallah center and two doctorate researchers specialized in psychopathology. Each domain and item was reviewed for structure and clarity, redundant inquiries eliminated, and ambiguous wording modified.

#### 4.3.3 Concurrent validity

The questionnaire and subscales were significantly and highly positively correlated with another established measure of family support scale (Dunst et al., 1986) [52]. The questionnaire was significantly associated (p < 0.01) with family support scale (r = 0.40).

#### 4.3.4 Internal consistency

Cronbach's α reliability coefficients were calculated for all the subscale and composite scores. Table (2) shows the results.

**Table (2):** Cronbach's Alpha coefficients.

Dimension	Cronbach's Alpha	N of Items
The first dimension	.763	7
The second dimension	.903	7
The third dimension	.883	6
The fourth dimension	.805	5
total degree	.879	25

Table (2) shows the reliability coefficients value of all the dimensions of the questionnaire are all high scores approaching the correct one. The total degree of reliability is (0.879) which is high value and approaching the correct one. It refers to the validity of the questionnaire for the application and the reliability of its results.

## 5 Results

**The first question:** What is the role of the family in supporting people with mental disorders?

To answer this question we used mean, standard deviation, and the rank for each item as the following:

The results presented in Table (3) show the mean, standard deviation, and rank of seven different items related to the role of families in supporting individuals with mental disorders. The study highlights the importance of family support for individuals with mental disorders, as families play a crucial role in helping individuals with mental disorders integrate into society and recover.

**Table (3):** The means and standard deviation for the first dimension.

No	Item	Mean	Std. Deviation	Rank
1	The family works to develop the skills, abilities, and desires of the person with a mental disorder	3.70	.871	6
2	The family must admit the wishes of the person with a mental disorder and examine what they can do	3.69	.719	7
3	Not feeling ashamed of the patient, taking him on social occasions and helping him to integrate into society	4.48	.469	2
4	Create a stable family and emotional atmosphere, helping the patient to recover	4.41	.532	3
5	Find new appropriate ways to treat the patient with mental disorder and his health	3.95	.845	4
6	The family works to provide support and assistance to the patient	4.49	.931	1
7	Avoid blaming the patient and notifying him that he has failed	3.86	.907	5
General mean		4.08	0.75	

The highest-ranked item in this study was “The family works to provide support and assistance to the patient” (mean = 4.49), indicating that participants considered this to be the most important aspect of family support. This finding is consistent with previous research that has shown that family support is critical in improving outcomes for individuals with mental disorders.

Another highly ranked item was “Not feeling ashamed of the patient, taking him on social occasions, and helping him to integrate into society” (mean = 4.48). This result suggests that families can help individuals with mental disorders overcome the stigma associated with mental illness by providing them with opportunities to participate in social activities.

The item “Create a stable family and emotional atmosphere, helping the patient to recover” (mean = 4.41) was also highly ranked, indicating that family support is not only important for helping individuals with mental disorders integrate into society but also for their recovery. This finding highlights the critical role of families in creating a supportive and nurturing environment for individuals with mental disorders.

In contrast, the items “The family works to develop the skills, abilities, and desires of the person with a mental disorder” (mean = 3.70) and “The family must admit the wishes of the person with a mental disorder and examine what they can do” (mean = 3.69) were ranked lower. These results suggest that participants may not consider these aspects of family support to be as important as the other items. However, it is important to note that these items may still be important in certain contexts, and further research is needed to understand their relative importance.

Overall, the general mean of 4.08 indicates that participants considered family support to be important in supporting individuals with mental disorders. These results have important implications for mental health professionals and policymakers who should recognize the critical role that families play in supporting individuals with mental disorders and develop policies and programs that support family involvement in the treatment and recovery process.

**The second question:** What does the family need to cope with the needs of a person with a mental disorder?

To answer this question we used a mean, standard deviation, and the rank for each item as the following:

The results in Table (4) show the importance of providing appropriate care and support for people with mental disorders. The study identified seven key factors that can contribute to the well-being of people with mental health issues.

**Table (4):** The means and standard deviation for the second dimension.

No	Item	Mean	Std. Deviation	Rank
1	Teaching basic skills that help people with mental disorders to move and develop in society	3.81	.922	6
2	Follow-up drug and psychological treatment with the patient	4.30	.938	1
3	Understand the disease, the conditions a patient can go through, and the symptoms that accompany the disease	3.82	.881	5
4	Professional help and guidance from specialists to adapt to the situation	4.22	.980	2
5	Providing a specialized center for patient care and family support	4.19	.925	4
6	Society accepts the patient and works to integrate it into the community	3.75	.858	7
7	Providing a suitable treatment that links the family's capabilities with the patient's psychological needs	4.22	. 931	3
General mean		4.04	0.92	

The top-ranked factor in the study was “follow-up drug and psychological treatment with the patient,” which indicates the crucial role of ongoing treatment and management of mental health conditions. This result highlights the importance of regular appointments with mental health professionals and the need for patients to stick to their treatment plan.

The second highest-ranked factor was “professional help and guidance from specialists to adapt to the situation,” which highlights the importance of mental health professionals in providing guidance and support to patients and their families. This result also underscores the significance of specialized centers that can provide comprehensive care and support to individuals with mental health issues.

Other important factors identified in the study include the need for society to accept patients and work to integrate them into the community, providing specialized centers for patient care and family support, and understanding the disease and its symptoms.

**The third question:** What is the importance of family rehabilitation for caring for a patient with a mental disorder?

To answer this question we use the mean, standard deviation, and the rank for each item as the following:

The most highly ranked item as presented in Table (5) is “The strong interaction between the family and the patient” with a mean score of 4.44, indicating that participants recognize the importance of family involvement in the treatment process. This finding is consistent with the idea that family members can play an important role in supporting the patient and monitoring their progress.

**Table (5):** The means and standard deviation for the third dimension.

No	Item	Mean	Std. Deviation	Rank
1	The strong interaction between the family and the patient	4.44	.898	1
2	Reduction of the burden of treatment costs through treatment centers and hospitals	4.31	.982	3
3	Confidence inside the patient, as the family is the source of confidence for the patient	4.05	.866	5
4	Cooperation with a psychotherapist to help the patient	4.29	.857	4
5	Rehabilitation helps relieve the family's psychological burden and enable them to accept the disease	3.57	.966	6
6	Rehabilitation works to understand the conditions of the disease and how to deal with the patient	4.35	.903	2
General mean		4.17	0.91	

Another important factor identified by the participants is the “Cooperation with a psychotherapist to help the patient” (mean score of 4.29). This highlights the importance of seeking professional help and guidance from mental health specialists to effectively manage mental disorders.

The study also reveals the importance of rehabilitation programs in understanding the conditions of the disease and how to deal with the patient, as well as relieving the family's psychological burden and enabling them to accept the disease (mean score of 4.35 and 3.57, respectively). These findings underscore the importance of providing comprehensive care and support for both the patient and their family.

**The fourth question:** What are the behaviors of family members that may increase or decrease stress for the patient?

To answer this question we used a mean, standard deviation, and the rank for each item as the following:

The results presented in Table (6) show that Item 3, “Bullying family members of the patient negatively affects him in treatment” has the highest mean score of 4.36 and is ranked number 1. This indicates that the respondents strongly believe that bullying family members have a negative impact on the patient's treatment. It is important for family members to be supportive and understanding during the patient's treatment to promote positive outcomes.

**Table (6):** The means and standard deviation for the fourth dimension.

No	Item	Mean	Std. Deviation	Rank
1	Failure of the patient to feel that it is a burden on the family, positively affecting the patient	3.94	.892	4
2	Building confidence in the patient helps in treating the patient	4.30	.910	2
3	Bullying family members of the patient negatively affects him in treatment	4.36	.880	1
4	Exposing the patient to the pressures of daily life affects him negatively	4.02	.820	3
5	Caring for the patient and good treatment affects the patient	3.88	.913	5
General mean		4.10	0.88	

Item 2, “Building confidence in the patient helps in treating the patient” has a mean score of 4.30 and is ranked number 2. This suggests that building confidence in the patient can be an effective way to treat them. A positive attitude can help the patient overcome challenges and improve their overall well-being.

Item 4, “Exposing the patient to the pressures of daily life affects him negatively” has a mean score of 4.02 and is ranked number 3. This item emphasizes the importance of protecting the patient from stressful situations during treatment. High levels of stress can negatively impact the patient's health and treatment outcomes.

Item 1, “Failure of the patient to feel that it is a burden on the family, positively affecting the patient” has a mean score of 3.94 and is ranked number 4. This item suggests that when the patient does not feel like a burden to their family, it can positively impact their treatment. Family members can play a critical role in making the patient feel comfortable and supported throughout their treatment.

Item 5, “Caring for the patient and good treatment affects the patient” has the lowest mean score of 3.88 and is ranked number 5. Although it has the lowest mean score, it is still an important factor in the patient's treatment outcomes. Providing good care and treatment can have a positive impact on the patient's well-being and recovery.

In conclusion, the table provides valuable insights into the role of family in the patient's treatment. It highlights the importance of providing emotional support, building confidence, and protecting the patient from stressors. Healthcare professionals can use this information to better educate and support families in providing care and treatment for their loved ones.

**The fifth question:** Are there statistically significant differences in the role of family members involved in supporting people with mental disorders due to the three variables: age, gender, and work?

### 5.1 First: age

One –way Anova test was used and Table (7) shows results:

**Table (7):** The differences in the role of family members involved in supporting people with mental disorders due to age.

	Sum of Squares	Df	Mean Square	F	Sig.
Between Groups	.063	2	.032	.242	.786
Within Groups	211.145	348	.131		
Total	211.209	350			

Table (7) shows that the results of the ANOVA indicate that there are no statistically significant differences in the role of family members involved in supporting people with mental disorders due to age. The F-value of 0.242 and significance level of 0.786 for between groups suggest that the differences between the means of the age groups are not statistically significant. Mental health professionals and policymakers should be aware that age alone may not be a significant factor in the level of support provided to people with mental disorders and consider other variables that may impact the role of family members in supporting them.

### 5.2 Second: gender

We use an independent sample T-test, and the answers are shown as:

that The results of the *t*-test in Table (8) indicate that there are statistically significant differences in the role of family members involved in supporting people with mental disorders due to gender. The t-value of 2.259 and significance level of 0.05 suggest that the mean level of support provided by female family members is significantly higher than the mean level of support provided by male family members.

**Table (8):** The differences in the role of family members involved in supporting people with mental disorders due to gender.

Gender	N	Mean	Std. Deviation	Sig.	T
Female	203	124.2420	.98266	0.00	2.259
Male	147	95.0962	.96050		

### 5.3 Third: work

We use an independent sample T-test, and the answers shown as:

The results of the *t*-test in Table (9) reveal that there are statistically significant differences in the role of family members involved in supporting people with mental disorders due to work status. The t-value of 6.178 and significance level of 0.00 suggest that there is a significant difference in the mean level of support provided by employed and unemployed family members. Specifically, the mean level of support provided by employed family members is significantly lower than the mean level of support provided by unemployed family members.

**Table (9):** The differences in the role of family members involved in supporting people with mental disorders due to work.

Work	N	Mean	Std. Deviation	Sig.	T
Employed	206	134.4532	1.43678	0.00	6.178
Unemployed	144	172.0764	1.34521		

## 6 Discussion

Studying the family's role in supporting children with mental disorders is essential to developing effective interventions that involve and support the family unit in promoting the child's mental health and well-being. To add to the literature in this area, the present study aimed to explore the role of families in supporting children with mental disorders in the Arab world, specifically in one of the Gulf Cooperation Council (GCC) countries Qatar.

Several findings emerged from the descriptive analyses. First, most of parents reported that, “"The family works to provide support and assistance to the patient” and “"Not feeling ashamed of the patient, taking him on social occasions, and helping him to integrate into society”. This finding is consistent with previous research that has shown that family support is critical in improving outcomes for individuals with mental disorders [53,54].

Second, most teachers reported the need for “"follow-up drug and psychological treatment with the patient,” and “professional help and guidance from specialists to adapt to the situation,” This is consistent with findings of others studies [55,56].

Overall, the results of this hypothesis suggest that a comprehensive approach to mental health care that involves ongoing treatment and support, professional guidance, and community acceptance and integration is necessary for the well-being of people with mental disorders. It is important to note that mental health conditions can vary widely, and individual needs should be considered when developing treatment plans.

Third, the results highlight the importance of a holistic approach to the treatment and management of mental disorders that involves family support, collaboration with mental health professionals, and comprehensive rehabilitation programs. By addressing the needs of both the patient and their family members, we can improve treatment outcomes and help patients achieve better mental health and quality of life. Rehaibitation needs is widely documented in literature [57,58].

Fourth, the findings suggest that the highest-ranked behavior that can increase or decrease stress for the patient is bullying by family members. This behavior can have a negative impact on the patient's treatment and recovery process, and it is essential to avoid it. Another behavior that can increase or decrease stress for the patient is building confidence in the patient. When family members provide emotional support and encouragement to the patient, it can positively impact their treatment and recovery process. This is consistent with previous results from other research [59,60].

Fifth, findings in this study reveal that age alone may not be a significant factor in the level of support provided to people with mental disorders and consider other variables that may impact the role of family members in supporting them. Gender and work status proved to have an effect on the role of family members involved in supporting people with mental disorders. Mental health professionals and policymakers should be aware that gender may play a significant role in the level of support provided to people with mental disorders and consider gender-specific interventions to enhance the support provided by male family members. Age, gender and work play a vital role in the way how family members support children with mental problem [[61], [62], [63], [64]].

The findings of this study have implications for mental health professionals and policymakers. It suggests that family members who are unemployed are more likely to provide greater support to their loved ones with mental disorders.

This study provides valuable insights into the importance of family support for children with mental disorders in the Arab world and the GCC. The study highlights the specific roles and strategies families can use to support their children's mental health needs. The study's findings can be used to develop culturally sensitive interventions that promote family involvement in the management of mental health problems.

## 7 Limitations

However, the study has some limitations that should be considered when interpreting the results. Firstly, the study recruited only Qatari mothers and fathers, which may limit the generalizability of the findings to other populations in the Arab world and the GCC. Secondly, the study relied on self-reported data, which may be subject to bias. Finally, the study only focused on four mental disorders, which may limit the generalizability of the findings to other mental health problems.

## Funding

The Open access funding is supported by Qatar National Library.

## Author contribution statement

Abdulnaser A. Fakhrou; Taha R. Adawi: Conceived and designed the experiments; Performed the experiments; Analyzed and interpreted the data; Wrote the paper.

Sara A. Ghareeb: Conceived and designed the experiments; Contributed reagents, materials, analysis tools or data; Wrote the paper.

Atef M. Elsherbiny: Performed the experiments; Contributed reagents, materials, analysis tools or data.

Maryam M. AlFalasi: Conceived and designed the experiments; Contributed reagents, materials, analysis tools or data.

## Data availability statement

The data that has been used is confidential.

## Declaration of competing interest

The authors declare that they have no known competing financial interests or personal relationships that could have appeared to influence the work reported in this paper.
